# Thank you to *The Lancet Regional Health - Southeast Asia’s* clinical and statistical peer reviewers in 2024

**DOI:** 10.1016/j.lansea.2025.100542

**Published:** 2025-02-10

Mohammed Aabdi

Dilaram Acharya

Olusoji Adeyi

Prashanth K Adiga

Ronal Surya Aditya

Kaosar Afsana

Mehmood Ahmad

Anisuddin Ahmed

Bilal Ahmed

Sheikh Mohammad Fazle Akbar

Mert Akyuz

Pooya Alaedini

Harold Alderman

Alec Alec Morton

Hayder Al-Kuraishy

Reem S Almaghrabi

Jalemba Aluvaala

Rafi Amir-ud-Din

Kanya Anindya

Rebecca Appleton

Rajeev Aravindakshan

Nimalan Arinaminpathy

Rajalakshmi Arjun

Kathryn Arnold

Amit Aryal

Augustine Asante

Ulla Ashorn

Rodrigo Abensur Athanazio

Sachin Atre

Charlotte Avanzi

Shally Awasthi

Chandrika Azad

Saleha Azeem

Yeji Baek

Bahador Bagheri

Emmanuel Baja

Ahad Bakhtiari

Jatinder Bali

Stefano Ballestri

Satchit Balsari

Abhay Bang

Indu Bansal

Jenny Barrett

Ian G Barron

Rebecca J K Bartlein

Alfred Bartolucci

Abhishekh Basavarajegowda

Buddha Basnyat

Anup Bastola

Saurav Basu

Priyam Batra

Himmatrao Bawaskar

Kirtti Bebarta

Rishikesh V Behere

Belaineh Girma Belaineh

Aniruddha Belsare

David Beran

Divya Bhandari

Omesh Bharti

Nishant Bhatia

Vineet Bhatia

Suraj Bhattari

Zulfiqar Bhutta

Jose Bines

Kare Birkeland

David Makram Bishai

Gunilla Björling

Batel Blechter

Hannah K Blencowe

Farid Boumédiène

Sofie Braet

Jessica I Bravo-Zúñiga

Clive Brown

Frederico Bruzzi de Carvalho

Atul Madhukar Budukh

Jane Burns

Sharath Burugina Nagaraja

Joaquim Bustorff-Silva

J A Cairns

Denton Callander

Andreina Carbone

Bianca Cata-Preta

Francesca Cavallaro

Mustafa Cesur

Li Chai

Achiraya Chaichaloempreecha

Debjit Chakraborty

Praveen Chandra

Andrew Chang

Pimlak Charoenkwan

Abhishek Chaturvedi

Ira Cheifetz

Chuqian Chen

Han Chen

Simiao Chen

Anish V Cherian

Wen-Hao Chiang

Delia Indira Chiarello

Tawanda Chivese

Karen Choong

Seema Chopra

Ranadip Chowdury

Eric Christensen

Diego Giulliano Destro Christofaro

Yashika Chugh

Hannah Clapham

Intira Collins

Andrew Copas

Arturo Corbatón-Anchuelo

Daniela Correia

Paul Crosland

Antoine Da-Costa

Ramesh B Daggubati

Sitasnu Dahal

Tina Damodar

Himanshu Dandu

Muhammad Daniyal

Mrinal Das

Sanchita Das

Taraprasad Das

Papa Dasari

Kevin M De Cock

Francesca De Felice

Edward Dee

Jason Deen

Gerco den Hartog

Smita Deshpande

Arnab Dey

Sanjit Dey

Thakur Dhakal

Neeraj Dhingra

Sameer Dhingra

Antonio Di Biagio

Nadia Diamond-Smith

Yingying Ding

Yuanling Lin Ding

Jino Distasio

Matthew Dodd

Kunchok Dorjee

Yeshey Dorjey

Thinley Dorji

Yanqiu Du

Manisha Dubey

Gaurav Raj Dwivedi

Zoe Dyson

Ferry Efendi

Aman Elwadhi

Chiranjeevi Kumar Endukuru

Paolo Eusebi

Christopher K Fairley

Ildar Fakhradiyev

Evandro Fei Fang

Marwa Farag

Eric Faragher

Forough Farrokhyar

Syeda Sadia Fatima

Rista Fauziningtyas

Junfeng Feng

Elizabeth M Fenton

Reza Forghani

Nermeen A Fouad

Anna Freni Sterrantino

Ryoma Fukuoka

Rahul Gajbhiye

Kurubaran Ganasegeran

Trivadi Ganesan

Shuvadeep Ganguly

Anjali A Ganpule

Suman Garai

Tushar Garg

Sneha Gautam

Nilesh Gawde

Nicholas Geard

Stefan Gebhardt

Sandeep Ghatak

Animesh Ghimire

Sushmita Ghoshal

Paramjit Gill

Surajit Giri

Prathiksha Giridharan

John Gleeson

Gyanendra Gongal

Catherine Goodman

Lallindra Viranjan Gooneratne

Maya Gopalakrishnan

Radhika Gore

Dipti Govil

Jagdish Goyal

Annalisa Grandi

Catherine Grant

Santiago Grau

Kathryn Greenwood

John Gregson

Harpreet Singh Grewal

Anneke Grobler

Humberto Guanche Garcell

Hao Guo

Abha Gupta

Bal Kishan Gupta

Himanshu Gupta

Parul Chawla Gupta

Rajeev Gupta

Ranjan Gupta

Salil Gupta

Sapna Gupta

Soumya Gupta

Emily Gurley

Ashutosh Halder

Raph L Hamers

Ariel Hammerman

Claudia Hanson

Roopa Hariprasad

Ingunn Harstad

Kenneth Harttgen

Matire Harwood

Rubayyat Hashmi

Divna M Haslam

Mateusz Hasso-Agopsowicz

Karen Elizabeth Hay

Ying He

Yulei He

Benjamin Hegarty

Sonia Hegde

Gonzalo Hernandez

Robert Heyderman

Henrike Heyne

Galib Muhammad Shahriar Himel

David Hipgrave

Erin Hobin

Christopher Holmberg

Md Alamgir Hossain

Yuanchao Hu

Bingsheng Huang

Jiunchi Huang

Nur Sabrina Idrose

Sundus Iftikhar

Claudio Intimayta Escalante

Ana-Maria Iosif

M Mofizul Islam

Sheikh Mohammed Shariful Islam

Sufia Islam

Karthikeyan Iyengar

Hari S Iyer

Bart Jacobs

Shuchi M Jain

Courtney Jarrahian

Kana Ram Jat

Afzal Javed

Benjarath Javed

Craig Jefferies

Masamine Jimba

William Joe

Jacob John

Noyal Joseph

Smita Joshi

Stefan Kabisch

Pradnya V Kakodkar

Nutan K Kamath

Mona Kanaan

Vijaya Kancherla

Suvi Kangas

Sonika Kanotra

Monica Kapasa

Sandip Kapur

Anindya Kar

Claire Karekezi

Asima Karim

Ganesan Karthikeyan

Kokouvi Kassegne

Sanin Kazi

Mark Kelson

Zoe Kelson

Emily Kendall

Rick Kennedy

Maryam Keshtkar-Jahromi

Anuradha Khadilkar

Himanshu Khajuria

Asaduzzaman Khan

Suliman Khan

Arun Kharat

Ali Khashan

David Kleiner

Mikashmi Kohli

Rajeev Zachariah Kompithra

Zisis Kozlakidis

Jesse Krakauer

Kailash Krishnan

Evangelos Kritsotakis

Prerna Kukreti

Anil Kumar

Jogender Kumar

Kaushal Kumar

Paninjukunnath Ravi Kumar

Pradeep Kumar

Praveen Kumar

R Krishna Kumar

Ranjeet Kumar

Sumit Kumar

Kumar Kumaran

Priyanka Kumari

Hannah Kuper

Anura Kurpad

Daniel M Lage da Cruz

Arnaud Laillou

Sham Lal

Carlos Laranjeira

Savita Lasrado

Matthew Laurens

Zhe Kang Law

Avula Laxmaiah

Christopher LeBoa

Jinkook Lee

Truls Michael Leegaard

Yiting Lei

Jie Li

Chun-Yu Lin

Siyuan Liu

Xiaoxue Liu

Rakesh Lodha

Christopher A Loffredo

Amos Loh

Watcharin Loilome

Manuel Lombardi

Janet Long

Imnameren Longkumer

Raúl López-Izquierdo

Chin-Li Lu

Shuang Lu

Tom Lung

Lola Madrid

Ajesh Basantharan Maharaj

Padkuduru A Mahesh

Pankaj Malhotra

Amyn Malik

Iram Malik

Kanika Malik

Mamunur Malik

Sara Mallinson

Luis Malpica Castillo

Jayanthi Maniam

Mohammad Ali Mansournia

Ben Marais

Paola Marchesini

Srinivas Marmamula

Homero Martinez

Glenn A Martínez

Basavaraj Mathapati

Umang Mathur

Kousaku Matsubara

Bozigar Matthew

Elizabeth McClure

Benjamin Joseph James McCormick

Mary McGowan

Barbara McPake

Shaffi Mdala

Esmaeil Mehraeen

Rachana Mehta

Satish Mendonca

Ramshekhar N Menon

Fiona Mensah

Jaume Mesquida

Edward Mills

Minja Milovanovic

Omid Mirmosayyeb

Vijay Kumar Mishra

Vikash C Mishra

Christina Mitropoulou

Hirokuni Mizoguchi

Viswanathan Mohan

Prasanna Simha Mohan Mohan Rao

Pratap Mohanty

M Rubaiyat Hossain Mondal

Jee Youn Moon

Bente Morseth

Arupendra Mozumdar

Vamshi Krishna Muddu

Abdul Razzaq Mughal

Abdifatah Muhummed

Ivan Müller

Hana Müllerová

Joko Mulyanto

Daniel Munday

Malaisamy Muniyandi

Manoj Murhekar

Godfrey Musuka

Ulrike Mütze

Naila Nadeem

David Naimark

Tatsuo Nakamoto

Luis Filipe Nakayama

Devaki Nambiar

Arindam Nandi

Tarun Narang

Angela Narayan

Nosheen Nasir

Gathari Ndirangu

Varshini Neethi Mohan

Hannelie Nel

Cassandra Nemzoff

Dinesh Neupane

Mai Nguyen

Huan Ning

Hiroshi Nishi

Atsushi Nohara

John Norrie

Bernard North

Ntwali Placide Nsengiyumva

Alexandre Nunes

Patrick Michael O'Connor

Owen O'Donnell

Shu Okugawa

Ioana Diana Olaru

Catherine Oldenburg

Tope Olubodun

David Ong

Shu Hwa Ong

Anu Oommen

Eric David Ornos

Assaf Oron

Isao Oze

Llewellyn Padayachy

Hirak Pahari

James Paik

Priyankar Pal

Jay Pan

Ramendra Pati Pandey

Raja Panwar

Nikolaos Papanas

Ken Kuljit Parhar

Patrizio Pasqualetti

Avanish Patel

Manish Pathak

Deepak Patil

Sumeet Patil

Muhammad Mainuddin Patwary

Jayter Paula

Rosanna Peeling

Javier Perez-Saez

Henry Perry

Olumuyiwa James Peter

Giuseppe Pezzotti

Prathip Phantumvanit

Georgina Phillips

Sirinya Phulkerd

Rene Pierpont

Manon Pigeolet

Ashwin Pillai

Neethi P Pinto

Michael Plank

R N Planken

Barry Popkin

Prasad D Pore

Jalandhar Pradhan

Rifky Octavia Pradipta

Sarbeswar Praharaj

Robin Prescott

Samuel J Pullen

Archana Purushotham

Saravanakumar Puthupalayam Kaliappan

Raoh Fang Pwu

Unaib Rabbani

Anton Rahardjo

A K M Fazlur Rahman

Mohammad Meshbahur Rahman

Rajesh Kumar Rai

Akhil Rajendra

Prasad Rajhans

Harsh Rajvanshi

Prema Ramachandran

Sainath Raman

Kim Ramasamy

Vasanthi Ramesh

Siddarth Ramji

Ketki Ranade

Kannan Ranganathan

Jaya Ranjalkar

Alok Ranjan

Fahimeh Ranjbar

Megha Rao

Didier Raoult

Ruwan Ratnayake

Abhishek Raut

Vikram Raut

Aravind Ravi Kumar

Ramu Rawat

Naeem Raza

Rehana Rehman

Joel Michael Reynolds

Mehr Muhammad Adeel Riaz

Henry Rice

Jane Louise Rich

Wachara Riewpaiboon

Carlos Rojas-Roque

Michele Romoli

Catherine E Ross

Jeremy Ross

Avijit Roy

Unmesha Roy Paladhi

Dorte Rytter

Swetha S Kumar

Gayatri Saberwal

Md Sabiruzzaman

Marzieh Saei Ghare Naz

Marc Saez

Muhammad Safdar

Pragyan Monalisa Sahoo

Arpita Sahu

Shovan Kumar Sahu

Nasir Salam

Naveen Salins

Sonali Pankaj Salvi

Sundeep Salvi

Martin Salzmann-Erikson

Rujipat Samransamruajkit

Leif Erik Sander

Benjarin Santatiwongchai

Milena Šantrić-Milićević

Swarup Sarkar

Mustafa Saroar

Rakesh Sarwal

Sujata Saunik

Akshar Saxena

Alyssa Sbarra

Edward Schenck

Frederic Schoenberg

Pietro Scicchitano

Giada Sebastiani

Wing Shan Queenie See

Kiruthika Selvaraj

Arjune Sen

Sirisha Senthil

Florina Serbanescu

Sujata Sethi

Yashendra Sethi

R Setiowati

Muhammed Shabil

Chintan Shah

Faraaz Shah

Sweta Shah

Noor Ahmad Shaik

Varun Shamanna

Rajasubramaniam Shanmugam

Arvind Kumar Sharma

Ashish Sharma

Nandini K Sharma

Pawan Sharma

Praveen Kumar Sharma

Priyanka Sharma

Rajesh Sharma

Sarit Sharma

Ali Sheidaei

Lumaan Sheikh

Kai Sheng Hsieh

Tahmina Shirin

Mark Shlomovich

Dhan Shrestha

Sujani Shroff

Zubin Cyrus Shroff

Sanne Sigh

Siham Sikander

Maggy T Sikulu-Lord

Thapat Silalertruksa

Betty Simon

Aditya Singh

Brijesh Singh

Deependra Singh

Pratibha Singh

Preet Amol Singh

Pushpendra Singh

Anju Sinha

Avik Sinha

Pranay Sinha

Sanjeev Sinha

Sanjay Sood

Bundit Sornpaisarn

Tayana Soukup

Parikipandla Sridevi

Padma Srivastava MV

Anca Stoian

Tor Strand

Raj Kumar Subedi

Narayana R Subramaniam

Paulomi Sudhir

Kunjbihari Sulakhiya

Kexin Sun

Peifang Sun

Naveen Sunder

Karin Sundström

Dev Sunuwar

Yam Sureau

Jyotsna Suri

Shoba Suri

Roshan Fakirchand Sutar

Sumathi Swaminathan

Saif Sayeed Syed

Padmavathyamma Narayanapillai Sylaja

Moizza Tahir

Vibhor Tak

Rayner Kay Jin Tan

Ajay Tandon

Rohit Tandon

Julian Tang

Sweefong Tang

Lili Tao

Shuman Tao

Roy Taylor

Rangaswamy Thara

Tun-Linn Thein

Linda Theron

Alvin G Thomas

Prasad Thota

Amanda Thrift

Tian Tian

Shiyam Sunder Tikmani

Zheng Quan Toh

Xunliang Tong

Stephanie Topp

Perihan Torun

Phyllida Travis

Rima Triatin

Vrijesh Tripathi

Feng-jen Tsai

Pham Tuan

Tumaji Tumaji

Pauline Turnbull

Roberto Umpierre

Budi Utomo

Efstratios Vakirlis

Cherian V Varghese

Puneet Verma

Mukul Vij

C R Vijay

Lakshmi Vijayakumar

Sheila Vir

To Nhu Vu

Nguyen Lam Vuong

Tabassum Wadasadawala

Nitya Wadhwa

Ralph Waesch

Naomi Waithira

Taiichi Wakiya

Dylan D Walters

Bingyi Wang

Jing Wang

Jingjing Wang

Oghenebrume Wariri

Anup R Warrier

Reynold G Washington

Paul Weiss

Christian Morberg Wejse

Franck Wieringa

Phoebe Williams

Yiu Ming Wong

Peter Wright

Chih-Da Wu

Luyu Xie

Xiaoxu Xie

Chander Prakash Yadav

Kapil Yadav

Uday Narayan Yadav

Gowri Yale

Pan-Chyr Yang

Zhiming Yang

Sandhya Kanaka Yatirajula

Pengpeng Ye

Eng-Kiong Yeoh

Medine Caliskan Yilmaz

Jianhai Yin

Dong Keon Yon

Aisha Yousafzai

Mohamed Zailani

Jeffrey Steven Zaltzman

Haijun Zhang

Zirong Zhao

Haidong Zou

